# IgA-producing B cells in lung homeostasis and disease

**DOI:** 10.3389/fimmu.2023.1117749

**Published:** 2023-03-01

**Authors:** Youri Bertrand, Alba Sánchez-Montalvo, Valérie Hox, Antoine Froidure, Charles Pilette

**Affiliations:** ^1^ Centre de Pneumologie, Otorhinolaryngologie (ORL) et Dermatologie, Institut de Recherche Expérimentale et Clinique, Faculté de Pharmacie et des Sciences Biomédicales, Université Catholique de Louvain, Brussels, Belgium; ^2^ Allergy and Clinical Immunology Research Group, Department of Microbiology, Immunology and Transplantation, Katholieke universiteit (KU) Leuven, Leuven, Belgium; ^3^ Department of Otorhinolaryngology, Cliniques Universitaires Saint-Luc, Brussels, Belgium; ^4^ Service de Pneumologie, Cliniques Universitaires Saint-Luc, Brussels, Belgium

**Keywords:** IgA^+^ B cells, lung B cells, lung mucosal immunity, upper airway immunity, airway disease

## Abstract

Immunoglobulin A (IgA) is the most abundant Ig in mucosae where it plays key roles in host defense against pathogens and in mucosal immunoregulation. Whereas intense research has established the different roles of secretory IgA in the gut, its function has been much less studied in the lung. This review will first summarize the state-of-the-art knowledge on the distribution and phenotype of IgA^+^ B cells in the human lung in both homeostasis and disease. Second, it will analyze the studies looking at cellular and molecular mechanisms of homing and priming of IgA^+^ B cells in the lung, notably following immunization. Lastly, published data on observations related to IgA and IgA^+^ B cells in lung and airway disease such as asthma, cystic fibrosis, idiopathic pulmonary fibrosis, or chronic rhinosinusitis, will be discussed. Collectively it provides the state-of-the-art of our current understanding of the biology of IgA-producing cells in the airways and identifies gaps that future research should address in order to improve mucosal protection against lung infections and chronic inflammatory diseases.

## 1 Introduction

IgA is the most secreted antibody isotype in the human body, mainly at mucosal surfaces where it plays protective roles such as immune exclusion. There are two subclasses of this immunoglobulin, namely IgA1 and IgA2. Compared to IgA1, IgA2 has a shorter hinge region and is therefore more resistant to pathogen-derived bacterial proteases (1, 2). There is only 10 to 20% of IgA2 in the blood (3), whereas this proportion increases in the lung mucosa, with approximately 25 to 30% of IgA2 in bronchoalveolar fluid (3, 4). In addition, monomeric IgA is predominant in serum in contrast with mucosal sites where there is mostly dimeric IgA (d-IgA) (80%) (2, 5). In the bronchus, epithelial cells express the polymeric Ig receptor (pIgR) which binds IgA and can be internalized to transport it to the lumen. The transcytosis ends with the cleavage of the C-terminal part of pIgR, the secretory component (SC), which is bound to d-IgA (6). The complex consisting of d-IgA and SC is called secretory IgA (S-IgA) and its main role is to prevent pathogens in the mucosal lumen from entering the body, which is called the immune exclusion (7–9). Beside pIgR, IgA can interact with cells through different receptors such as IgA Fc receptor I (FcαRI or CD89) or the transferrin receptor 1 (CD71) (10, 11). CD89 is the main one in human, expressed by myeloid cells: neutrophils, eosinophils, monocytes, macrophages and dendritic cells (11). Mice do not express CD89 (but retained to Fcα/μR, for both IgA and IgM), leading to the development of transgenic mice expressing the human CD89 to enable taking this interaction into account whilst studying the role of IgA *in vivo*, e.g. in (11)IgA nephropathy (12–14), cancer (15) or the inflammatory role of serum IgA (16).

IgA-producing B cells in the mucosa were mostly studied in the gut and much less in the airways, leading to gaps in knowledge for the later. This review aims to describe the different subsets of IgA^+^ B cells in the airways during homeostasis and their changes and roles following immunization and in disease such as IgA deficiency or chronic airway/lung diseases. It also summarizes the mechanisms underlying activation and homing of IgA^+^ B cells in the lung, as compared to the gut.

## 2 Phenotype and location of IgA^+^ B cells in the airways

### 2.1 Conventional B cells

**Figure 1:**
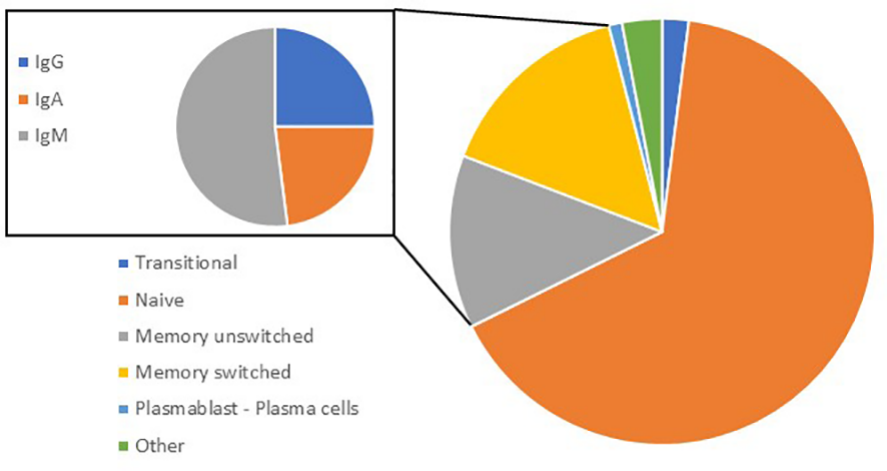
Proportion of B-cell subsets in blood. In peripheral blood, transitional (CD24^+^ CD38^+^) represent 2.4%; naive (CD27^-^; IgD^+^) 65%; memory unswitched (CD27^+^; IgD^+^) 15%; memory switched (CD27^+^; IgD^-^) 13; plasmablasts/plasma cells (CD24^-^; CD38^+^) 1%; and others 2.5%. Out of memory switched B cells (inset), 23% are IgG^+^, 21% are IgA^+^: 21%, and 52% are IgM^+^ (IgM^+^ only or IgM^+^/IgD^+^).

Currently identified B-cell subclasses have been mainly studied in blood and usually express CD45, CD19 and CD20. Naïve (IgD^+^/IgM^+^), transitional, memory unswitched (CD27^+^ in humans and IgM^+^), memory switched (CD27^+^, IgE^+^, IgG^+^ or IgA^+^) and antibody-secreting B cells (CD20^-^, CD38^+^ and CD138^+^) also known as plasma cells (PCs) are the main types of conventional (B2) B cells (17, 18). The majority (60-70%) of circulating/blood B cells are naive, expressing both IgD and IgM while IgA^+^ memory B cells (MBCs) represent ˜ 10% of B cells in the peripheral blood (19) (**Figure 1**). They are circulating and can relocate to secondary lymphoid organs (SLOs) such as the lymph nodes (LNs) or tertiary lymphoid organs (TLOs). In the lung, the mucosa associated lymphoid tissue is known as the inducible bronchus associated lymphoid tissue (iBALT) (20). iBALT is a lymphoid aggregate that is usually rarely detectable in the lung at homeostasis in mouse or in man, but can develop following infections or during chronic inflammation (20, 21).

### 2.2 Resident B cells

**Figure 2:**
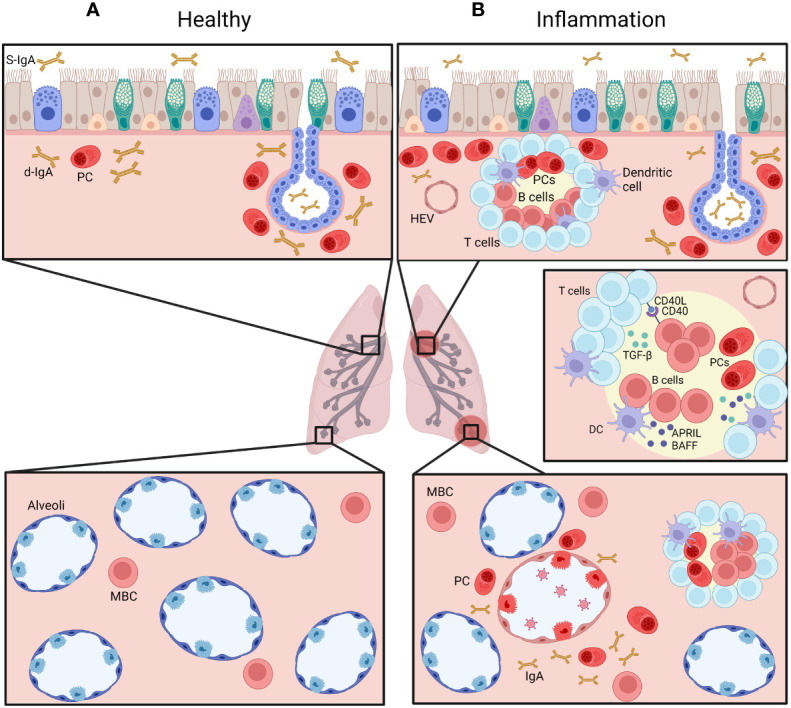
Distribution of IgA^+^ B cells in the airways, at homeostasis **(A)** and during chronic inflammatory condition **(B)**. Plasma cells (PC) are normally mainly found beneath the airway epithelium and in submucosal glands (*upper panels*), representing a pro-IgA niche, and secrete d-IgA, which is transported through pIgR-mediated routing to the mucosal lumen. IgA^+^ memory B cells are also found in the alveolar septa (*lower panels*). During chronic inflammation, accumulation of IgA^+^ PCs is observed, along increased numbers of IgA^+^ B cells within lymphoid follicles, following cognate interactions with T cells as well as innate signaling from dendritic cells (DC). Created with BioRender.com.

Although circulating B cells are present in the lung, other specific subtypes are represented in this organ. A subset of tissue-resident memory B cells (BRMs) has been identified in the lung in 2012 (22) and was described as the main producer of IgA in the respiratory system (23). This subset of B cells has mostly been studied in mice; they were identified by iv injection of labelled anti-CD45 antibodies followed by sorting and FACS analysis of B cells (24). In this study, non-circulating cells represented almost half of B cells in the lung (24, 25). Even though a specific marker for this subset was not established (26), mouse BRMs express CD69 and CD103 (22, 24, 26, 27) (**Table 1**). A larger proportion of BRMs are switched to IgA as compared to circulating blood B cells (24). Their presence and ability to stay (and not recirculate) in the lung is probably due to an increased expression of CXCR3, a receptor to the CXCL11 chemokine (23, 24). In addition, CXCR3 KO mice do not have BRMs and display an impaired production of IgA in the lung (23). BRMs cells have been described in different locations in the lung; after immunization against influenza virus, BRMs were observed in iBALT (28, 29) while alveolar BRMs were also reported after a similar immunization to *influenza* (29) or *Streptococcus pneumoniae* (29, 30) (**Figure 2**). Barker et al. also identified a putative subset of BRMs in the lungs from human donors, where most memory B cells (CD27^+^) expressed CD69 while naive B cells (IgD^+^) did not (30). Moreover, most of these CD69^+^ B cells were class-switched and did not express CD38, indicating that they were not yet differentiated into plasma cells (30).

**Table 1:** Comparison of IgA^+^ B cells in airways and in the gut.

	LUNG		GUT	
	Human	Mouse	Human	Mouse
**Homing**				
Adhesion	α4β1 integrins/VCAM1 CD62L/PNAd LFA1	α4β1 integrins/VCAM1 CD62L/PNAd LFA1	α4β7 integrins/MAdCAM1 CD62L/MAdCAM1	α4β7 integrins/MAdCAM1 CD62L/MAdCAM1
Chemokine receptor - chemokine	CXCR5 – CXCL13 CXCR3 – CXCL11	CXCR5 – CXCL13 CXCR4 – CXCL12 CCR7 - CXCL19 CCR7 - CXCL21	CCR9 – CCL25 CCR10 – CCL28	CCR9 – CCL25 CCR10 – CCL28
**B-cell subsets**				
Memory (MBC)				
Markers	CD23^low^ CD62L^low^ CD27^+^ CD38^+^ CD69^+^ CD80^+^ CD103^+^ CXCR3^hi^	CD23^low^ CD62L^low^ CD38^+^ CD69^hi^ CD103^hi^ CXCR3^hi^	CD27^+^ CD38^+^ CD40^low^ CD62L^-^	B220^+^ CD23^+^
Expected function	Long-lived cell with specific antibodies maturation; differentiation into PCs after challenge	Long-lived cell with specific antibodies maturation; differentiation into PCs after challenge	Long-lived cell with specific antibodies maturation; differentiation into PCs after challenge	Long-lived cell with specific antibodies maturation; differentiation into PCs after challenge
Resident (BRM)				
Location	iBALT or alveoli	iBALT or alveoli	GALT, Peyer patches and lamina propria	GALT, Peyer patches and lamina propria
Plasma (PC)				
Markers	CD20^-^ CD27^+^ CD38^+^ CD138^+^	CD20^-^ B220^+^ CD27^+^ CD38^+^ CD138^+^ CXCR3^+^	CD20^-^ CD19^+^ CD27^+^ CD38^+^ CD138^+^	α4β7-integrin^+^ CD73^+^ PD-L2^+^ CD80^+^
Expected function	Secretion of specific antibodies	Secretion of specific antibodies	Secretion of specific antibodies	Secretion of specific antibodies
Long-lived (LLPC)	Not described	Not described	Subset: CD19^-^ CD45^-^ CD27^hi^ CD38hi CD138^+^ Subset: CD19^+^ CD45^-^ CD27^hi^ CD38^hi^ Subset: CD19^+^ CD45^+^ CD27^hi^ CD38^hi^ Blimp1 IgA^hi^	B220^-^ CD38^+^ CD138^+^
**Breg**				
Markers	Immature subset: CD24^+^/CD38^+^ Memory subset: CD24^+^/CD27^+^ Plasmablast subset: CD27^+^/CD38^+^ B10 subset: CD25^+^/CD71^+^ B1 subset: CD43^+^	Immature subset: CD24^+^/CD38^+^ Memory subset: CD24^+^/CD27^+^ Plasmablast subset: CD27^+^/CD38^+^ B10 subset: CD25^+^/CD71^+^ B1 subset: CD43^+^	Immature subset: CD24^+^/CD38^+^ Memory subset: CD24^+^/CD27^+^ Plasmablast subset: CD27^+^/CD38^+^ B10 subset: CD25^+^/CD71^+^ B1 subset: CD43^+^	Immature subset: CD24^+^/CD38^+^ Memory subset: CD24^+^/CD27^+^ Plasmablast subset: CD27^+^/CD38^+^ B10 subset: CD25^+^/CD71^+^ B1 subset: CD43^+^
Expected function	Regulation of immune response; secretion of IL-10, IL-35 and TGF-β	Regulation of immune response; secretion of IL-10, IL-35 and TGF-β	Regulation of immune response; secretion of IL-10, IL-35 and TGF-β	Regulation of immune response; secretion of IL-10, IL-35 and TGF-β
**B1**				
Markers	Not described	CD19^+^ IgD^low^ CD23^+^ CD5^+^ and CD5- subsets	Not described	CD19^+^ B220^low^ IgD^low^ CD43^+ ^ CD25+ CD73^+^PDL2^+^ CD5^+^ and CD5-subsets
Expected function	Not described	Production of cross-reactive natural antibodies Clearance of apoptotic debris Modulation of inflammation	Not described	Production of cross-reactive natural antibodies Clearance of apoptotic debris Modulation of inflammation
**Isotype (and isoform)**	Dimeric IgA1, mainly (70%; IgA2, 30%)	Dimeric IgA	Dimeric IgA2, mainly (60%)	Dimeric IgA
**MALT**	Not readily detectable in the healthy lung iBALT induced by infection/inflammation	Not readily detectable in the healthy lung iBALT induced by infection/inflammation	Well developed in healthy individuals CD11 cells in lamina propria	Well developed in healthy individuals CD11 cells in lamina propria

### 2.3 Plasma cells

Plasma cells (PC) are the terminal differentiation stage of the B-cell lineage that secretes antibodies. They differentiate from GC B cells or memory B cells and are CD27^+^, CD38^+^ and CD138^+^ but CD20^-^ (18). In the mucosa, IgA is the most represented isotype (70% to 90%) (31, 32), most of them (around 80%) being located in the gut, especially in the gut-associated lymphoid tissue (GALT) (32, 33). A recent research of 3 control subjects suggested that IgA1 is the predominant subclass among IgA PCs in healthy lungs (two thirds of IgA^+^ PCs) (34), while IgA2 increases during the late stage of COVID infection (34).

As most IgA^+^ PCs are located in the gut, IgA-secreting PCs are also present in the murine lung. Twenty days after primary *influenza* virus infection or intranasal vaccination, IgA^+^ PCs derived from BRMs can be observed in the submucosal areas, beneath the bronchial epithelium (23, 29). Interestingly, IgA^+^ PCs are already detectable 4 days post-secondary infection, next to the alveoli instead of more proximal airways (29). IgA^+^ PCs have also been described in the lung after challenging ovalbumin (OVA)-sensitized mice (35).

A unique subset of IgA^+^, long-lived PCs (IgA^+^ LLPC), able to survive for years, was identified in the bone marrow (36, 37). Further studies led to identify that bone marrow IgA^+^ PCs were Ly6a^hi^ TIGIT^-^ and had a particularly high capacity to produce antibodies and a BCR being highly mutated due to SHM in the GC (38). It was thus suggested that IgA^+^ LLPC are differentiating in the mucosa before recirculating and relocating to the bone marrow. Interestingly, IgA^+^ LLPC were also observed in the gut, 80% of LLPCs being located in GALT (33, 39). Gut and bone marrow LLPCs share a long-lived phenotype (B220^-^), the same antibody repertoire and somatic hypermutation rate (40). The gut predominance as source of those cells could be explained by the fact that the gut represents the main source of overall IgA^+^ B cells. In addition, more attention has been given to gut-derived rather than lung-derived LLPCs (e.g., IgE^+^ LLPCs). However, one study showed that a subset of IgA^+^ LLPCs was induced upon intranasal vaccination or local allergen challenge (ovalbumin) that was more efficient than systemic sensitization (35, 41). Thus, the condition of induction and exact role of lung-derived IgA^+^ LLPCs remain unclear and should be further studied especially in human. (**Table 1**).

### 2.4 Regulatory B cells

In blood as well as in tissue, there are several subsets of B cells that can be classified as Bregs because of their regulatory role; these include CD24^+^/CD27^+^ with both a memory and regulatory phenotype; CD24^+^/CD38^+^ immature cells, CD27^+^/CD38^+^ plasmablasts as well as a subset of CD25^+^/CD71^+^ B cells and CD43^+^ B1 cells. Bregs may modulate inflammation, notably *via* cytokine secretion of IL-10, IL-35 or TGF-β (42). A recent study discovered a subset of blood-derived IgA^+^ Bregs that produce IL-10 also expressing PD-L1 (43). Interestingly, whereas those IgA^+^ Bregs expressed IL-10, PD-L1 and Fas-L, they did not express the classical Breg markers, suggesting that IgA^+^ Bregs may represent a new subset (**Table 1**). Another study identified a subset of circulating IgA-secreting Bregs in mice that was induced following intranasal exposure to a fusion protein made of flagellin (FlaA) and Bet v1 allergen (44). They showed production of both IgG and IgA by those Bregs. Whereas those IgA antibodies could not bind to the triggering antigen, it was demonstrated that IgA^+^ Bregs could inhibit Th2 inflammation, opening therapeutic perspectives in allergy (44).

### 2.5 B1 cells

B1 cells have been discovered in mice as the main subset of B cells found in the pleural and peritoneal cavities (45). Considered as part of the innate immune system, B1 cells are developed at neonatal stages and have the ability to secrete antibodies prior to any infection or antigen exposure (45, 46). B1 cells from these cavities are characterized by their expression of CD19, surface IgM (high), surface IgD (low), CD43 and CD11b (47, 48). This population can be further divided into two main phenotypes, namely B1a cells (CD5^+^) and B1b cells (CD5^-^) (49). In the pleural cavity, the majority of B1 cells (75%) are corresponding to the B1a phenotype (48) but those cells may trans-differentiate to B1b cells after stimulation by pathogens (48, 50). Both B1 subsets have the ability to produce “natural antibodies” (NAbs) (49), which can be IgM but also IgG3 or IgA isotype. NAbs can be defined as non-specific antibodies synthesized prior to immunization (51, 52). They are cross-reactive and detect a large spectrum of antigens with low affinity, including self-antigens (51, 53) and a hypothesis even suggests that NAbs rise following exposure to self-antigens (53). Because of their cross-reactivity, NAbs secreted in the mucosa can interact with potential pathogens even during the first encounter with the microorganism and play a key role in the innate immune response to infections (54–56). For instance, anti-GAL NAbs are observed in serum since childhood and are able to bind to several microorganisms (57) and anti-phosphorylcholine NAbs recognize Gram-positive bacteria (46). In addition, it has been shown that their low affinity autoreactivity can play a helpful, homeostatic role in, notably with anti-phosphoryl NAbs that can bind to apoptotic cell membranes (46, 58).

Although there is no consensus yet for specific markers of B1 cells in the human body (52, 53), some “B1-like cells” have been described and characterized as CD27^+^, CD43^+^, CD70^-^ in peripheral blood and in the umbilical cord (46, 53, 59). A review on the ontogeny of human B1 cells highlighted that this subset represents 1% of B cells in the adult blood, this number decreasing with age (60). When looking specifically at IgA-producing B1 cells, a population expressing CD27 and CD43 was described in blood (61, 62). Moreover, a particular phenotype of IgA^+^ innate lymphocytes named “natural helpers” was observed in both gut and lung (63–66). Researchers discovered that those natural helpers were producing cytokines similar to Th2 such as IL-5, IL-6 and IL-13 (64, 65). In the lung, these natural helpers have been only characterized in mouse models, with a potential role in allergic asthma (65). (**Table 1**).

## 3 Mechanisms of homing and priming of IgA^+^ B cells in the lung

### 3.1 Homing of IgA^+^ B cells

B cells circulating in the bloodstream can enter tissues, either in lymph nodes (LN) or in mucosa associated lymphoid tissue (MALT) (33, 67, 68). Although the generic mechanisms beyond the homing to those locations are similar, some specific axis are also involved. The homing of B cells has been well described in the gut; in contrast, there are many differences with the iBALT. The iBALT is rarely observed in healthy individuals, their induction requiring the presence of inflammatory signals (20, 69). In response to IL-17, different stromal cells present at the early stage of iBALT formation, such as follicular dendritic cells, can recruit B cells (70, 71). This recruitment starts in high endothelial venules (HEVs), at the border of iBALT, where B cells circulating in the bloodstream can interact with endothelial cells to home into this tissue. Several phenotypes of circulating B cells (naïve, transitional, memory unswitched, memory switched) express L-selectin (CD62-L), a receptor to peripheral lymph node addressin (PNAd) that is a signalling molecule on the membrane of endothelial cells in iBALT (67). This leads to the attraction of B cells to the walls of HEVs, allowing the adhesion of B cells to the endothelium *via* α4β1 integrins interacting with V-CAM1 (67). This is an important difference compared to the homing into GALT, where the adhesion is driven by the binding of α4β7 integrins to Mad-CAM1 (67). Subsequently, the CXCL13 chemokine secreted by DCs in the central zone will attract B cells through their CCR5 receptor, leading to the formation of a B-cell follicle in the iBALT (20, 70). It has been described that in some iBALT structures, fibroblast-like cells could alternatively guide B cells *via* the CXCL12-CCR4 axis (71).

The development of mucosal vaccines is a real challenge, with homing of B cells to the lung being an important element of this research. Back in 2004, it was proven that after intranasal inoculation of SARS-CoV virus, mice developed pulmonary lymphoid follicles along with upregulation of the chemokines CXCL9, CXCL10 and their receptor CXCR3 (72). Similar results were observed when mice were intranasally inoculated with *Bordetella bronchiseptica* (73). Moreover, transgenic mice lacking CXCR3 had fewer lymphocytes in lung tissue and displayed a delayed clearance of the bacteria (73). More recently a murine model of intranasal immunization with *Influenza* virus demonstrated an increase of B cells in the lung as well as higher levels of CXCL11 and IgA (22, 23). The studies showed that the IgA-producing B cells in those murine models were also expressing CXCR3 and that this receptor was a key for local IgA production, as CXCR3^-/-^ mice had fewer IgA^+^ cells in the lung (22, 23). A study conducted on delta-inulin, a potential adjuvant, showed that pulmonary immunization against *Influenza* coupled with this adjuvant led to a 4-fold increase of the number of CXCR3^+^ class-switched memory B cells in the lung as well as an increase of local IgA and IgG production (74). When re-challenged by a lethal exposure to *Influenza*, mice that did not receive the adjuvant during the immunization suffered from weight loss and poor survival after 8 to 9 days while mice who received the adjuvant were fully protected (74). Collectively these studies in murine models suggest that the homing of B cells *via* CXCR3 pathway is key to the immune response to infection and could be targeted for improving the development of mucosal vaccines.

### 3.2 Regulation of lung B cells towards IgA

**Figure 3:**
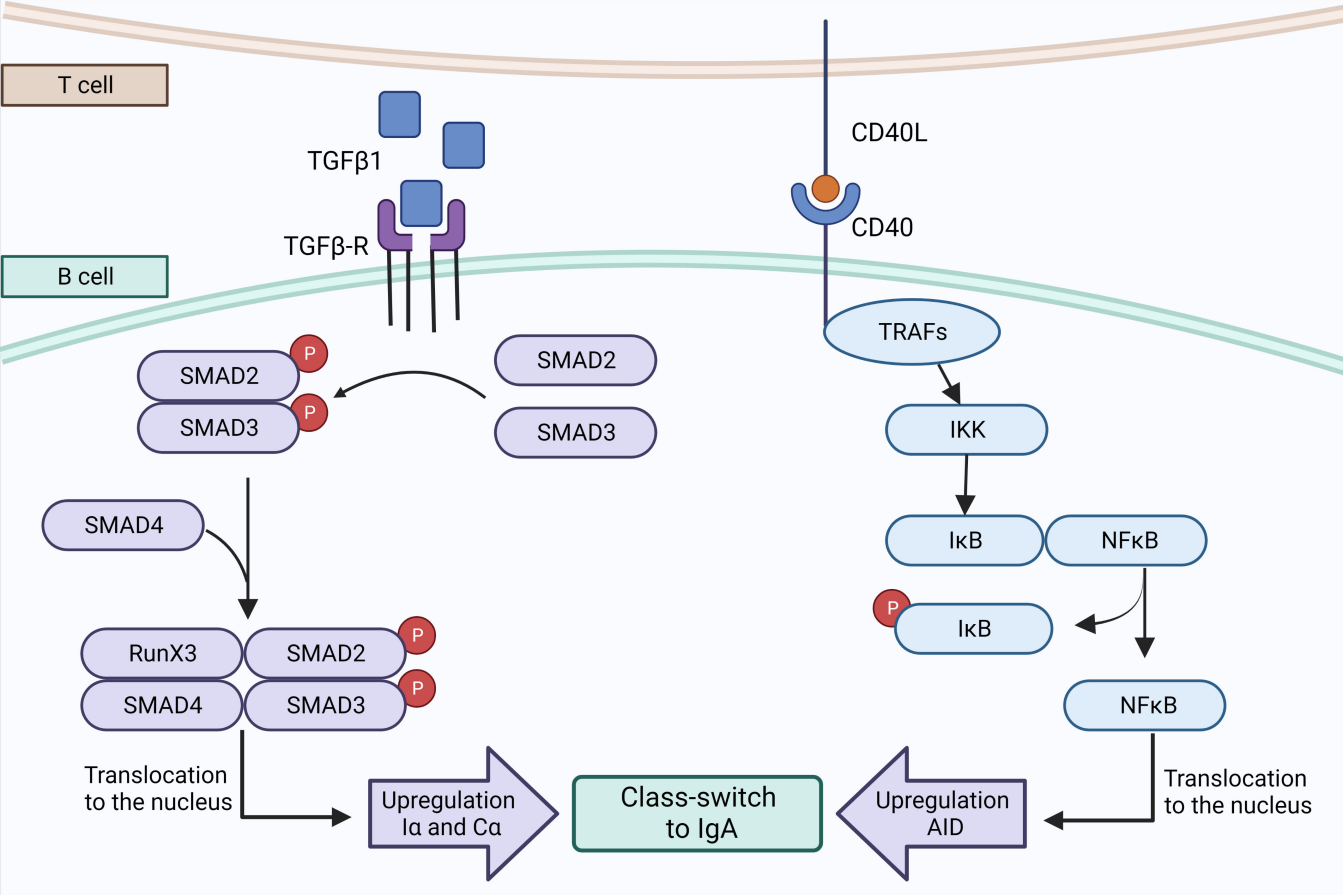
T cell-dependent pathway to IgA synthesis. Th cells may secrete active TGFβ1 which binds to TGFβ-R, triggering the phosphorylation and dimerization of Smad2 and Smad3 that are interacting with Smad4 and RunX3 to promote the transcription of Iα and Cα. The second signal consists of CD40 that interacts with CD40L expressed by T cells, to recruit a complex of TRAFs protein that activates IκB kinase. The phosphorylation of IκB by IKK unleashes NFκB and allows its translocation to the nucleus where it promotes the transcription of AID and CSR to IgA. Created with BioRender.com.

It is known that a class-switch recombination (CSR) to IgA can happen *via* T-cell dependent or independent pathways (33, 75–77). Since 1989, transforming growth factor β1 (TGF-β1) has been identified as the main cytokine driving CSR of naive B cells to IgA by a T-cell dependent pathway (78–80) (**Figure 2**). In the lymph nodes of the lungs or the gut, more specifically in the germinal center (GC), naive B cells can be activated when their BCR binds to their cognate antigen. Whenever this event happens, the expression of CCR7 will increase at the expense of the expression of CXCR5. Thus, those B cells will be attracted by CCL21, at the periphery of the GC, where CD4^+^ Th cells reside (77, 81). Th cells can secrete active TGF-β1, which binds to TGF-β receptor (TGF-βR) and activates its serine/threonine kinase activity (75, 82), leading to the phosphorylation and activation of SMAD2 and SMAD3 and allowing them to interact with SMAD4 and RunX3 to promote the expression of Iα and Cα, which are mandatory for the initiation of CSR (75, 80, 83). In addition, CD40 ligand (CD40L), a protein from the tumor necrosis factor (TNF) family also expressed by Th cells, is mandatory for the T-cell dependent pathway (84) (**Figure 3**). CD40L can bind to CD40, a transmembrane protein of B cells which will induce the recruitment of TNF receptor associated factor (TRAF) followed by activation of the NF-κb kinase inhibitor (IKK) (75, 85), inducing its phosphorylation and subsequently its ubiquitination to allow NF-κB to play its transcription factor activity and promote the transcription of *AICDA* (75, 85, 86). Other factors can synergize with CD40L, such as IL-4 which is able to activate the transcription factor STAT6 which also promotes *AICDA* transcription (86). The process of T-cell dependent CSR toward IgA takes a week (75) and can lead to different endpoints; B cells can return to the follicle, proliferate and undergo somatic hypermutation (SHM), or recirculate in the periphery after differentiation into MBCs or ASCs (77).

**Figure 4:**
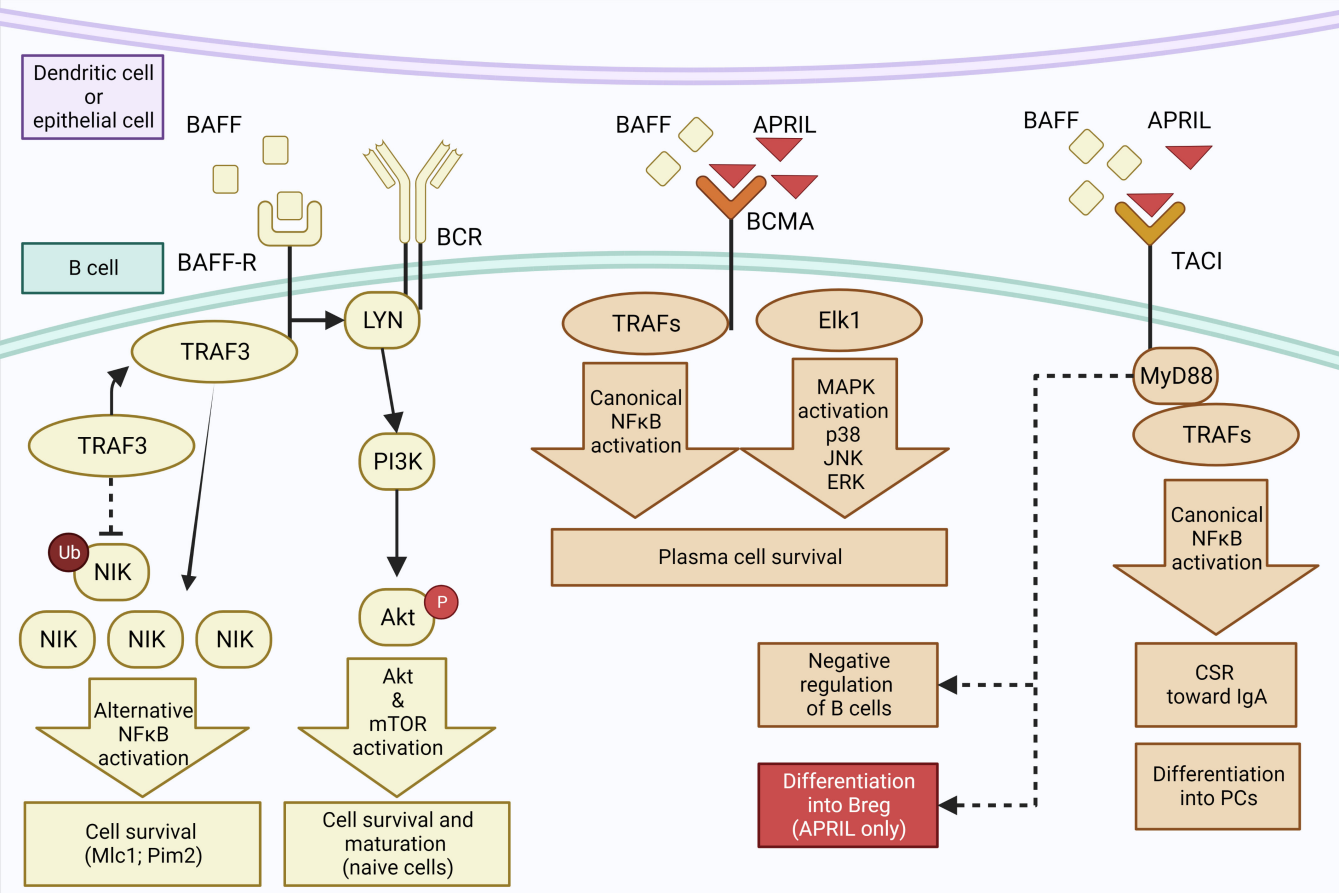
T cell-independent pathway to IgA synthesis. Epithelial and dendritic cells may secrete BAFF that binds to BAFF-R, inducing an interaction between BAFF-R and TRAF3 which induces ubiquitinylation of NIK. Accumulation of NIK leads to activation of NFkB that promotes B-cell survival. BAFF-R also interacts with BCR and Lyn, activating the PI3 kinase and Akt/mTOR pathways to promote survival and maturation of naive B cells. Secondly, BCMA receptor can also bind BAFF as well as APRIL, inducing the recruitment of TRAFs and the activation of NFkB as well as of Elk1 and MAP Kinase pathways, promoting the survival of plasma cells. Third, ligation of TACI by BAFF or APRIL activates the canonical NFkB pathway *via* its interaction with MyD88, leading to the differentiation of B cells into PCs and class switch to IgA. It may also induce a negative regulation of B cells through an unknown mechanism, or the differentiation of B cells into Bregs when the binding by APRIL occurs in the absence of BAFF. Created with BioRender.com.

T cell-independent pathways allow B cells to switch to IgA faster and can be mediated by dendritic cells (DCs) (75, 76, 87) (**Figure 4**). It has been shown that lung DCs are able to induce a local CSR toward IgA with a better efficiency than spleen DCs (88). The main cytokines secreted by DCs that induce IgA switching are B-cell activating factor (BAFF, or BLyS) and A proliferation-inducing ligand (APRIL) (76), two cytokines of the TNF superfamily (89). Both are ligands to the B-cell maturation antigen (BCMA) and transmembrane activator et CALM interactor (TACI) (90), whereas BAFF may also bind a selective BAFF-receptor (BAFF-R) (90).

TACI receptor has slightly stronger affinity for BAFF than APRIL (91) but it is not able to bind all forms of those molecules; only oligomeric forms of APRIL or BAFF can bind to TACI (92). Interestingly, TACI is able to induce survival signal but also to negatively regulate B cells (92). The pathways behind these opposite activities are not fully understood but TACI can interact with MyD88, an activator of NF-κB (43, 93) as well as with TRAF3 that inhibits the NF-κB pathway (77). The interaction between TACI and BAFF and APRIL can induce the CSR to IgA but also the differentiation into PCs by promoting the expression of *Prdm1* (93), which encodes Blimp-1 that is a key transcription factor promoting XPB1 and inducing a secretory phenotype (94).

It has been reported that ligation of TACI by APRIL in the absence of BAFF, also induces CSR to IgA (5). Although the mechanisms of CSR driven by TACI remain unclear, TACI could interact with the MyD88 protein adaptor in order to activate NF-κB, inducing CSR by promoting the transcription of activation induced cytidine deaminase (*AICDA)* or C_H_ genes which are crucial for CSR (93). In addition, a recent *in-vitro* study showed that APRIL stimulation of TACI, coupled with IL-21 and in the absence of BAFF or TGF-β, induces the differentiation of B cells isolated from PBMC to a subset of Bregs expressing IgA and IL-10 (43). This study also suggested that this specific subset of IgA^+^ Bregs could play a protective role against experimental autoimmune encephalomyelitis or arthritis (43). A similar cell phenotype was observed in mice after immunization against an allergen, and induction of IgA^+^ Bregs in this context was also dependent on MyD88 and NFkB activation (44). Signalling pathways underlying the differentiation of B cells into IgA^+^ Bregs could open the way for new therapeutic strategies in allergic and inflammatory airway diseases.

BCMA is the second TNF receptor able to bind both BAFF and APRIL, with a stronger affinity for the later (95). This receptor plays a key role in the survival of PCs, a defect of BCMA or its ligand resulting in reduced number of PCs in the bone marrow (96). The signalling pathways downstream of BCMA remain unclear but it is clear that it cannot interact with Myd88 (97)and that they lead to survival signalling pathways such as the classical NF-κB, Elk-1, c-Jun N-terminal kinase, and p38 mitogen-activated protein kinase pathways (97, 98). BAFF can also bind with a strong affinity to BAFF-R, which is present on all B-cell subsets (99). This will induce the activation of both the canonical and alternative NF-kB pathways, leading to the induction of survival signals for B cells. In addition, ligation between BAFF and BAFF-R causes the activation of phosphatidyl-inositol 3 kinase (PI3K), which activates the downstream signalling pathway of Akt and also induces survival of B cells.

Retinoic acid (RA), a widely studied molecule derived from vitamin A, can interact with specific nuclear receptors expressed in various organs (100). In the lung, RA plays an important role for prenatal lung organogenesis (100) and supports post-injury alveolar regeneration in the adult lung (101). In addition to interactions with epithelial or mesenchymal cells, it also contributes to maintenance of mucosal immunity (102–105). Thus, DCs can synthesize RA which interacts with B cells *via* their RA-receptor (106). It has been demonstrated *in vitro* that RA induces a class-switch to IgA in B cells from human tonsils (106) and murine spleen by interacting with RA-receptors on B cells (107). In addition, RA may accelerate the differentiation process from B cells to PCs (108), further increasing the production of IgA antibodies. This is collectively supported by the fact that a defect in vitamin A results in impaired generation of IgA^+^ B cells (109).

## 4 Roles of IgA^+^ B cells in lung mucosal defense and in disease

Although selective IgA deficiency (IgAD) is the most prevalent immune deficiency, affecting 1 out of 100 to 1000 individuals and that most IgAD subjects are asymptomatic, this defect increases the risk of upper and lower respiratory tract infections (110). Studies in mice showed that although naive IgA^+/+^ and IgA^-/-^ mice have the same susceptibility against *influenza* infection (111–113), whenever they were exposed to *influenza* virus 5 weeks after vaccination without adjuvant, the survival of IgA -/- mice was decreased compared to controls, suggesting that IgA could provide postvaccinal protection (113). This statement is reinforced by the fact that local immunization against *influenza* induces the secretion of IgA by PCs as well as BRMs in submucosal areas of the airways and offers a better protection against infection than systemic immunization, which does not affect IgA levels (23). It is however known that IgA^-/-^ mice display abnormalities in generating other isotypes such as IgE or IgG (114). Nevertheless, intranasal vaccination that promotes S-IgA responses has gained interest, notably following COVID pandemics.

Chronic obstructive pulmonary disease (COPD) is a common respiratory disease and the third cause of death worldwide since 2019 (115). It is associated with tobacco exposure and characterized by emphysema and obstruction of small airways (116). Lymphoid aggregates are developing near the distal airways of COPD patients (117) and our group described that IgA^+^ B cells in those areas were the only ones that upregulate during disease, mostly in severe patients (118). An accumulation of IgA1 was also observed in the subepithelial area (119), consistently with an increased synthesis combined with the lack of transport to the lumen through the pIgR which is downregulated in COPD (120). In addition, IgA autoantibodies against cytokeratins 18 and 19, expressed by epithelial cells, are increased in the plasma from COPD patients (121). Activation of B cells in this disease could be mediated by T cells, and more specifically by IL-21-secreting Th17 cells within lymphoid follicles (118). It is also possible that airway epithelial cells contribute to activate B cells as primary bronchial epithelial cells from COPD patients provide signals (such as IL-6) to B cells to promote their IgA synthesis, possibly through TACI upregulation (119, 122).

Patients with cystic fibrosis (CF) are particularly susceptible to airway and lung infections. CF is a genetic autosomal recessive disease due to a mutation on the gene encoding for thecystic fibrosis transmembrane conductance regulator (CFTR) protein, a ion channel for chloride anions (123), leading to dehydration of the airway mucus that becomes sticky and promotes respiratory infections with opportunistic pathogens such as *Pseudomonas aeruginosa* (*Pa*) (124). In CF lungs, lymphoid aggregates that include IgA+ PCs (CD138+) were observed (124). Similarly, increased levels of IgA can be observed in serum and bronchoalveolar lavage (BAL) from CF patients compared to healthy controls (125). In addition, the increase in IgA in serum and airway secretions was correlated with chronic infection by *Pa* (126–128). These results were similar to murine models, where it was also demonstrated that *Pa* infection leads to an upregulation of pIgR in both control and CF mice as well as an increased production of IgA, leading to an increase of BAL S-IgA (126). It was also reported that BAFF mediates the induction of IgA following *Pa* infection in mice (129), whereas depletion of BAFF increases the susceptibility to *Pa* of CF mice. Thus, it seems that Pa infection induces a specific IgA immune response that is associated in CF with a global upregulation of the IgA/pIgR system, which relate notably to BAFF and IL-17 signalling. This response is not associated with the eradication/clearance of the pathogen but rather accompanies chronic infection.

In addition, IgA can also represent a biomarker of infection or disease severity in CF. It is long known that CF patients display elevated levels of autoantibodies, among which IgA autoantibodies, in serum and sputum (130, 131). It can be already observed in pediatric cohorts but the levels of some specific antibodies increase with the age (132–134). The most studied IgA autoantibodies in CF are antineutrophil cytoplasm antibodies (ANCA) and more specifically the one targeting bactericidal/permeability-increasing protein (BPI) (132, 135–138). Neutrophils are major immune cells present in CF airways, putatively playing protective roles against pathogens (139) while also exacerbating airway damage, e.g. by releasing neutrophil elastase that degrades peribronchial tissue (139). In addition, dead neutrophils are the main source of extracellular DNA that further thickens the mucus of CF patients (140). A meta-analysis on the topic indicates that around 50% of the patients had elevated levels of BPI-ANCA IgA (137). They also demonstrated that the levels of BPI-ANCA were associated with increased prevalence of *P. aeruginosa* infection, increased response to *P. aeruginosa* infection and decreased lung function (137). IgA autoantibodies directed against double-stranded DNA were also observed both in serum and sputum from CF patients but only the systemic one was associated with a worse outcome (140). Finally, IgA autoantibodies against PAD-4 were identified, also in serum and sputum secretions, correlating with disease severity (141). Furthermore, it was reported that serum levels of IgG BPI-ANCA correlated those of antibodies against *P.aeruginosa* (132)whereas these IgG autoantibodies are not present in the airways where only IgA BPI-ANCA are observed, despite that IgG antibodies against *P.aeruginosa* are also present, suggesting different pathways underlying local/airway vs systemic autoimmune responses (132). In another work in children with CF, an increase in the number of B cells was reported in blood compared to BAL, also supporting this hypothesis (142). Finally, a study on the response to *P.aeruginosa* infection in children demonstrated differences between systemic and local responses (143), all children with CF -whether they had chronic, acute, or even no history of *P. aeruginosa* infection- displaying higher BAL levels of IgA against *P. aeruginosa* than did control children. On the other hand, children with no history of *P. aeruginosa* infection had similar levels of serum specific IgA as control children (143). Thus, whereas IgA may play a protective role against infection, it is likely that it also play roles during autoimmune responses in the lung. Whereas IgA autoantibodies are well documented in CF, the presence of autoreactive B cells and their location or biology has been less studied. The relationship between a specific subset of natural killer T cells and IgG autoreactive B cells in the lung was explored in a murine model, showing that the number of germinal centers autoreactive IgG^+^ cells in the lung was inversely correlated to this T cell subset (144). To our knowledge, this has not been evaluated with IgA autoreactive B cells, probably mostly due to difficulties in isolating or detecting antigen specific B cells particularly in tissues where a method has however been developed using biotinylated specific antigens revealed by avidin-cyanin 3 (145).

Finally, the IgA transport system seems also altered in this disease (116, 146), with upregulation of pIgR expression allowing increased transcytosis of IgA into mucosal secretions (118, 137). despite an intrinsic inhibitory effect of the mutated CFTR on pIgR expression in cells or animals (126, 146).. When CFTR-mutated mice were infected with *P.aeruginosa*, pIgR expression was upregulated, probably through the IL-17 pathway, indicating that infection could restore and even upregulate the IgA/pIgR system in this disease (126).

In asthma, an association was recently described between disease severity and increased blood IgA^+^ B cells in a cohort of 154 patients and 28 healthy individuals (147). Using flow cytometry, this study showed that more severe asthma was associated with a decrease of circulating naïve and transitional B cells and an increase of IgA^+^ B cells compared to controls as well as compared to patients with mild asthma (147). It was also described that BAFF levels were increased in lung tissue and BAL (but not in serum) in human asthma (148, 149). In ovalbumin (OVA)-induced allergic experimental asthma (95), it was shown that those mice had increased IgA levels in both serum and BAL (150). Interestingly, eosinophils, which are major effector cells of allergic asthma, have a high expression of the myeloid IgA-receptor FCαR1 in mice (150), as observed in allergic patients (151). S-IgA can readily induce the degranulation of eosinophils (152, 153), suggesting that IgA could contribute to the pathogenesis of mucosal inflammation in asthma (150). Furthermore, inhibition of CD40-CD40L or OX40-OX40L interactions in mice results in lower total and OVA-specific IgA levels in lung tissue, BAL and serum, which could suggest that this increase is at least partly due to a T–cell-dependent mechanism (150).

IgA and IgA^+^ B cells could also play a role in both autoimmunity and fibrogenesis in interstitial lung disease. In particular, idiopathic pulmonary fibrosis (IPF), a deadly pathology characterized by the destruction of the architecture of the distal lung with an accumulation of collagen due to the hyperactivation of fibroblasts (154, 155). Recent data suggest that B cells and IgA could play a role in this pathology. First, lymphoid aggregates were observed in IPF lungs (156, 157) and elevated concentration of IgA antibodies in serum from patients with IPF has been associated with a decreased survival (158). IPF patients also display increased circulating IgA autoantibodies compared to healthy individuals (159, 160), which correlate with the number of TLOs in the lung (160). In addition, increased numbers of PCs and transitional B cells were present in the IPF lung tissue, and TLOs were strongly stained for IgA (159). Moreover, an increase of CXCL13 was observed in blood as well as in the lung tissue from IPF patients, particularly in TLOs and near the fibroblast foci (161). Furthermore, B cells in TLOs express CXCR5 (161) but not Ki67 (157), suggesting that they could mainly be recruited from blood through a CXCL13-CXCR5 axis. It is known that TGF-β is increased in IPF tissue (162), probably produced by Th cells in TLOs (159), leading to the hypothesis that CSR to IgA could be induced locally. The precise role of IgA^+^ B cells in IPF remains however unclear and should be further studied.

## 5 Roles of IgA^+^ B cells in upper airways

Few data exist on the role of IgA and IgA-producing B cells in sinonasal diseases (163). In patients with selective IgAD, 48-78% display recurrent upper airway infections, including rhinitis and rhinosinusitis (164–166), likely due to the major role of IgA against viral infections (167). Upper airway infections with viruses such as rhinovirus (168), influenza (169) and SARS-CoV2 (170) lead to an increase of S-IgA in the nasal lavage, which plays a protective role in man (169) and mice (126, 127). Additional evidence emerged from studies in endurance athletes, strenuous exercise decrease upper airway S-IgA production that correlates to the increased rate of upper respiratory tract infections (171–173).

In patients with allergic rhinitis (AR), nasal allergen challenge induces local IgA production (153), and specific nasal IgA responses have been reported for different allergens, including house dust mites (174), grass (175), ragweed (176, 177), birch pollen (178, 179), and red cedar (180) with antigen-specific IgA levels correlating with nasal symptoms (180). However, patients with IgAD are more likely to develop AR (165, 166) and AR patients have lower total IgA levels in the serum as well as in their nasal tissue compared to healthy controls (181, 182). One study showed that intranasal administration of ragweed-specific IgA protected against allergic inflammation in sensitized mice (183), and that the production of allergen-specific IgA in neonatal mice prevented the development of cockroach allergy (183). Several studies also reported that successful allergen immunotherapyis associated with allergen-specific IgA responses (184–191) along increased IgA^+^ B cells, PCs, and Bregs that correlate with the clinical benefit (192, 193).

In chronic rhinosinusitis (CRS) patients it has been reported that 16.7% has low levels of serum IgA, with 6.2% matching the definition of selective IgAD (194). Similarly CRS is more frequently reported in individuals with IgAD (up to 78%) compared to individuals with normal IgA levels (166). In patients suffering from CRS with nasal polyps (CRSwNP), increased numbers of B cells and PCs are found in polyp tissue (195, 196) and these PCs abundantly produce Igs including IgA. It has been reported that these B cells infiltrating NPs are contributing to organize TLOs (196–198), suggesting a unique B cell activation environment within NPs that is distinct from classic GC-mediated mechanisms (199). Several studies also reported overexpression BAFF in patients with CRSwNP (200–203) and of local IgA^+^ PCs (204), IgA1^+^ B cells serving as precursors for IgA2^+^ B cells (205). One recent paper also suggested the possibility of local conversion of antigen-specific IgA to IgE (206), but this needs to be validated. Increased S-IgA levels were observed in CRSwNP compared to healthy controls in one study (204), but not in another (207). The latter study also showed increased tissue IgA in CRSwNP with paradoxically reduced IgA antibody levels to *S. aureus* enterotoxin B (207), a powerful pro-Th2 superantigen. Elevated levels of IgA autoantibodies to double-stranded DNA and basement membrane proteins have also been observed in CRSwNP (208).

## 6 Conclusion

In the lung, IgA-producing B cells and IgA^+^ PCs are found in the airways, close to the surface epithelium and around submucosal glands, as well as in the lung parenchyma. Following its transport across the epithelium through pIgR-mediated trafficking, IgA antibodies protect the conducting airways *via* immune exclusion of inhaled pathogens and antigens. During disease state such as infections or COPD, iBALT can develop and recruits B cells by producing the chemokine CXCL13 that attracts the CCR5-expressing B cells. T cells that are present in iBALT can induce class-switching to IgA, through TGF-β and concomitant interaction between CD40L and CD40. Dendritic cells may also induce IgA synthesis in the lung by secreting APRIL and/or BAFF that bind to three different receptors and lead to the activation of NF-κB and Akt pathways that underlie survival and maturation of naïve B-cells. Interestingly, TACI activation is closely related to class-switching to IgA and differentiation into PCs.

Lung-tissue resident memory B cells were identified in the lung, but within airways and alveoli. Upon antigen rechallenge, those resident cells can move near the new infection site *via* CXCL13-CCR5 interaction, where they differentiate into PCs to secrete protective antibodies. This host response was not observed upon systemic immunization, suggesting that mucosal vaccines could represent a specific route for prevention against respiratory infections. In addition, a subset of Bregs that produce IgA was discovered following activation by APRIL in the absence of BAFF, or following activation by a specific fusion protein containing an allergen. Importantly, IgA^+^ Bregs produce IL-10 and are potent regulators of allergic inflammatory responses in mice. In contrast, IgA and IgA^+^ B cells could also contribute to chronic inflammation, possibly through autoimmune mechanisms. iBALT from COPD and IPF patients include IgA^+^ B cells, a feature that correlates with increased levels of circulating IgA autoantibodies directed against KRT18 and KRT19 (in COPD) or citrullinated proteins (in IPF). In the upper airways, IgA^+^ B cells may also play different roles, as patients with IgA deficiency are more susceptible to develop allergic rhinitis or CRS on the one hand, while in CSRwNP IgA autoantibodies against double stranded DNA could be detrimental on the other.

There remain many gaps in knowledge regarding IgA immunity in the lungs. Some of them are the characterization of B1-like and LLPC subsets, the selective pro-IgA signalling operating in the lung (including molecular signalling, e.g. behind TACI), as well as the regulation by IgA of airway/lung microbiome. Most importantly, future research should also address the functional role of IgA^+^ B cells that are present in several inflammatory diseases in the airways, to determine whether, how, and under which circumstances they exert protective and/or deleterious roles.

## Author contributions

YB drafted the manuscript. AS and VH wrote the upper airway chapter. AF revised the lung chapters. CP revised the manuscript. All authors contributed to the article and approved the submitted version.
